# Linking Hypothermia and Altered Metabolism with TrkB Activation

**DOI:** 10.1021/acschemneuro.3c00350

**Published:** 2023-08-08

**Authors:** Okko Alitalo, Gemma González-Hernández, Marko Rosenholm, Piia Kohtala, Nobuaki Matsui, Heidi Kaastrup Müller, Wiebke Theilmann, Anders Klein, Olli Kärkkäinen, Stanislav Rozov, Tomi Rantamäki, Samuel Kohtala

**Affiliations:** † Laboratory of Neurotherapeutics, Drug Research Program, Division of Pharmacology and Pharmacotherapy, Faculty of Pharmacy, University of Helsinki, Helsinki 00014, Finland; ‡ SleepWell Research Program, Faculty of Medicine, University of Helsinki, Helsinki 00014, Finland; § Center for Translational Neuromedicine, Faculty of Health and Medical Sciences, University of Copenhagen, Copenhagen DK-2200, Denmark; ∥ Department of Psychiatry, Weill Cornell Medicine, New York, New York 10021, United States; ⊥ Faculty of Pharmacy, Gifu University of Medical Science, 4-3-3 Nijigaoka, Kani, Gifu 509-0293, Japan; # Translational Neuropsychiatry Unit, Department of Clinical Medicine, Aarhus University, Aarhus N 8200, Denmark; ∇ Novo Nordisk Foundation Center for Basic Metabolic Research, University of Copenhagen, Copenhagen DK-2200, Denmark; ○ Department of Drug Design & Pharmacology, University of Copenhagen, Copenhagen DK-2100, Denmark; ◆ School of Pharmacy, University of Eastern Finland, Kuopio 70210, Finland; ¶ Afekta Technologies Ltd., Kuopio 70210, Finland

**Keywords:** antidepressant, energy metabolism, hypothermia, physiology, rapid-acting antidepressant, sedation, sleep deprivation, neuroplasticity

## Abstract

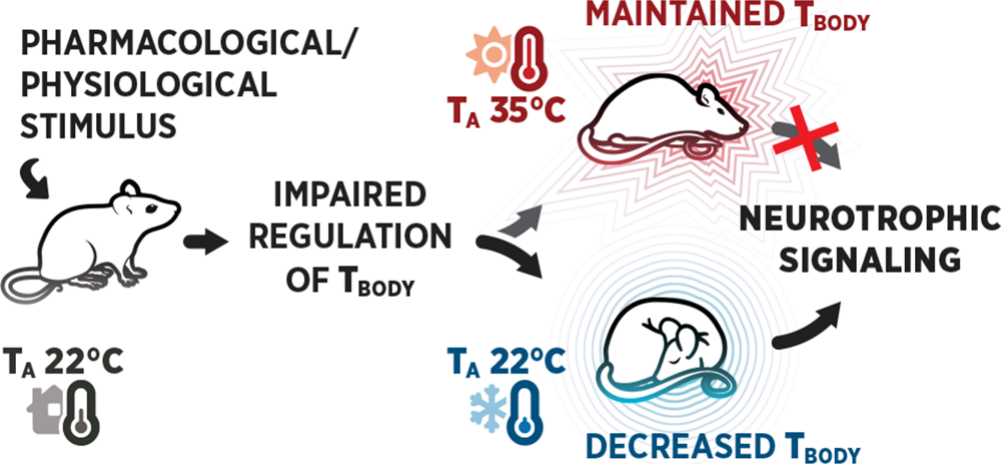

Many mechanisms have been proposed to explain acute antidepressant drug-induced activation of TrkB neurotrophin receptors, but several questions remain. In a series of pharmacological experiments, we observed that TrkB activation induced by antidepressants and several other drugs correlated with sedation, and most importantly, coinciding hypothermia. Untargeted metabolomics of pharmacologically dissimilar TrkB activating treatments revealed effects on shared bioenergetic targets involved in adenosine triphosphate (ATP) breakdown and synthesis, demonstrating a common perturbation in metabolic activity. Both activation of TrkB signaling and hypothermia were recapitulated by administration of inhibitors of glucose and lipid metabolism, supporting a close relationship between metabolic inhibition and neurotrophic signaling. Drug-induced TrkB phosphorylation was independent of electroencephalography slow-wave activity and remained unaltered in knock-in mice with the brain-derived neurotrophic factor (BDNF) Val66Met allele, which have impaired activity-dependent BDNF release, alluding to an activation mechanism independent from BDNF and neuronal activity. Instead, we demonstrated that the active maintenance of body temperature prevents activation of TrkB and other targets associated with antidepressants, including p70S6 kinase downstream of the mammalian target of rapamycin (mTOR) and glycogen synthase kinase 3β (GSK3β). Increased TrkB, GSK3β, and p70S6K phosphorylation was also observed during recovery sleep following sleep deprivation, when a physiological temperature drop is known to occur. Our results suggest that the changes in bioenergetics and thermoregulation are causally connected to TrkB activation and may act as physiological regulators of signaling processes involved in neuronal plasticity.

## Introduction

Accumulating evidence suggests that both classical and rapid-acting antidepressant drugs act by promoting synaptic plasticity in the adult brain. A core molecular mechanism of plasticity is the signaling of tropomyosin-related kinase B (TrkB), which acts as the cognate receptor for the brain-derived neurotrophic factor (BDNF).^1^ Pharmacologically diverse antidepressants, including tricyclic antidepressants (e.g., amitriptyline), selective serotonin reuptake inhibitors (e.g., fluoxetine), and rapid-acting antidepressants (e.g., ketamine; *N*-methyl-d-aspartate receptor [NMDAR] antagonist), converge in the activation of TrkB and its downstream signaling cascades, which include activation of the mammalian target of rapamycin (mTOR) and inhibition of glycogen synthase kinase 3β (GSK3β) in the adult rodent brain.^2−5^

Several mechanisms have been proposed to govern the antidepressant-induced TrkB activation. Initially, chronic administration of conventional antidepressants was shown to enhance BDNF expression in several brain areas.^6^ Subsequent research suggested that ketamine induces rapid BDNF translation and release.^2,7^ Certain drugs, like amitriptyline and 7,8-dihydroxyflavone, have also been proposed to act as direct agonists of TrkB.^8,9^ However, as many in vitro studies have proven unreliable in demonstrating convincing pharmacodynamic interaction of the drugs and TrkB receptor, there have been calls to reevaluate whether these drugs actually bind to the receptor in vivo at all and whether the observed downstream signaling is initiated in a canonical manner.^10−12^ Another more recent hypothesis suggests that antidepressants facilitate neuronal responsiveness to BDNF by binding to a novel transmembrane site of TrkB.^13^ Casarotto and colleagues propose that rapid-acting antidepressants bind to TrkB more readily than traditional antidepressants, which require chronic administration and accumulation in the brain tissue in order to reach TrkB signaling-promoting concentrations. However, earlier findings demonstrate that the intraperitoneal administration of conventional antidepressants prominently activates brain TrkB receptors rapidly within an hour^4,5^ and without any involvement of BDNF.^12^

To complicate the interpretation of past research further, the effects of some clinically effective antidepressants are problematic to explain using the principles of conventional receptor pharmacology. For example, according to our recent findings, nitrous oxide (N_2_O, “laughing gas”)^14^ and flurothyl—considered “a chemical electroconvulsive therapy” (ECT),^15^ as it produces epileptiform activity and significant electroencephalography (EEG) slowing during the subsequent postictal state—converge in activating TrkB signaling only after their acute pharmacological effects have dissipated.^16^ Although both flurothyl and N_2_O are eliminated from the body within minutes and have no active metabolites, TrkB becomes and remains phosphorylated long after the drug delivery.^16^ These findings suggest that the treatments render TrkB active through a mechanism that does not involve activity-dependent BDNF release and/or direct binding of the drug ligand to TrkB.

TrkB activation induced by flurothyl and N_2_O temporally coincides with the emergence of EEG slow-wave activity (SWA),^16^ a neurophysiological signature of deep sleep^17^ and reduced brain energy utilization^18^ and a biomarker associated with therapeutic efficacy of ECT.^19,20^ Notably, sleep deprivation (SD)—another nonpharmacological rapid-acting antidepressant^21^—also increases SWA during the following bout of sleep.^22^ Furthermore, there appears to be a strong association with activation of TrkB signaling and the potency of EEG suppression produced by several anesthetic agents, including putative rapid-acting antidepressant isoflurane^23−25^ and subanesthetic ketamine.^26^ Similar to earlier findings on NMDA receptor antagonists,^27^ we have observed in rodents how EEG slowing—induced by subanesthetic ketamine, N_2_O, and flurothyl—emerges gradually after their acute effects begin to fade out.^16^ Therefore, these findings have raised the possibility of an association between reduced electrophysiological activity and neurotrophic signaling.^28−30^

Collectively, emerging evidence suggests that antidepressant-induced TrkB activation may not involve a straightforward ligand release and/or receptor binding mechanism but could instead be associated with some components of the physiological state evoked by the pharmacological treatment.^31^ This response, besides being characterized by EEG slowing and neurotrophic signaling, co-associates with other physiological changes connected with deep slow-wave sleep: behavioral immobility,^32^ attenuated brain energy expenditure,^33^ and reduced body temperature.^34^ Here, we present evidence from molecular studies in mice showing that a range of pharmacological agents possessing sedative properties can activate neurotrophic signaling irrespective of their primary pharmacological target. We further demonstrate that similar mechanisms may be associated with physiological processes such as sleep, metabolism, and thermoregulation. This work provides new insights into the physiological changes underlying TrkB activation and urges to examine the antidepressant signaling from the perspective of brain physiology instead of being solely a conventional ligand-receptor interaction.

## Results

### Various Antidepressants, Sedatives, and Anesthetics Increase TrkB Phosphorylation

**Figure 1:**
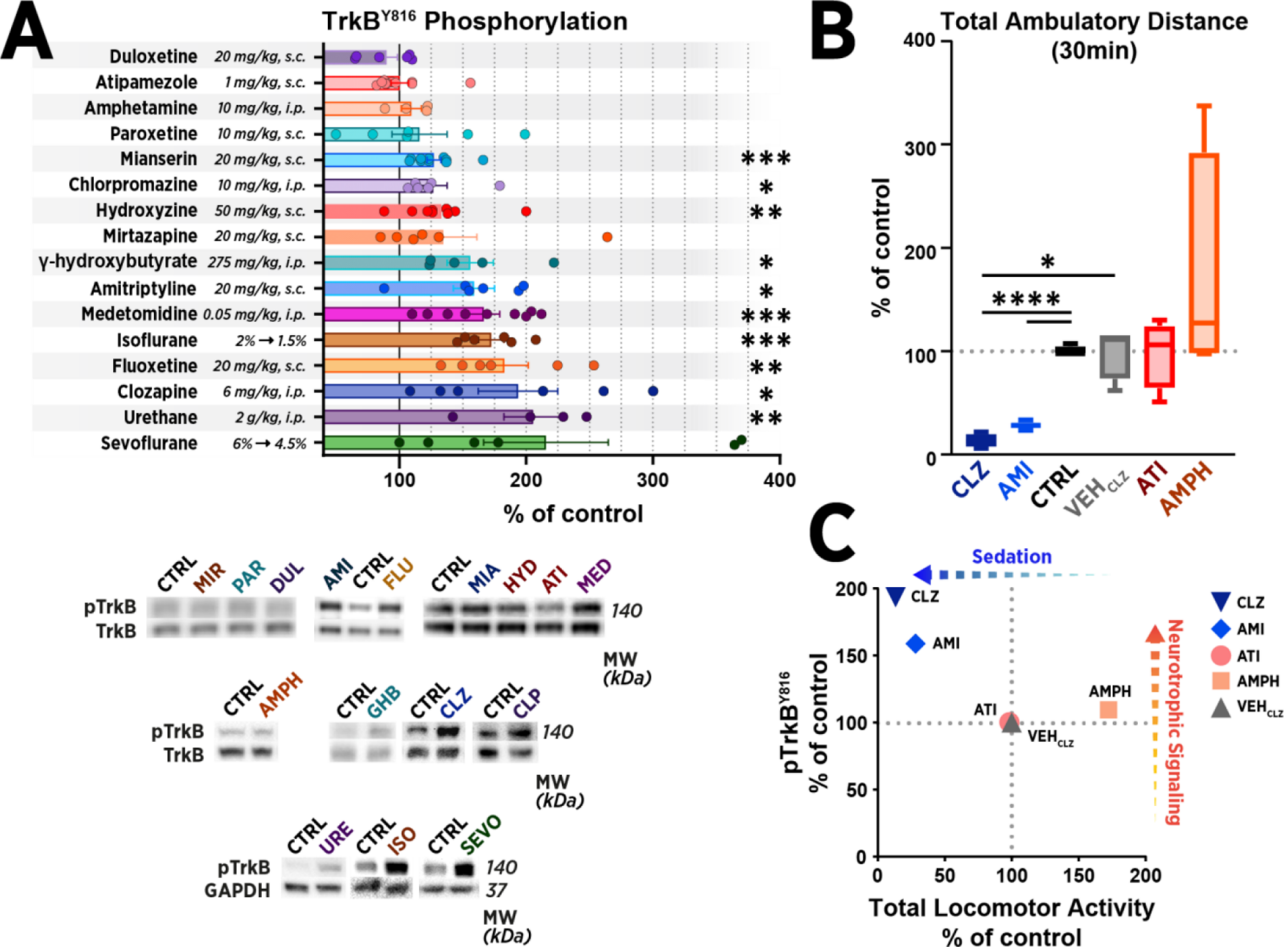
Pharmacological manipulation of TrkB signaling in the adult brain. (A) Diverse sedative-anesthetic agents increased the phosphorylation of TrkB^Y816^ in the medial prefrontal cortex of adult mice, whereas nonsedative antidepressants such as paroxetine and stimulant drugs such as amphetamine (AMPH) and atipamezole (ATI) showed a negligible effect. Phosphoproteins were normalized against corresponding total protein signal and compared to the control group (CTRL) set to 100%. Control animals for injected pharmacological agents were injected with a vehicle solution at an identical volume and route of administration, while control animals for isoflurane and sevoflurane were subjected to pressurized room air. See the Methods section for a detailed description of the treatments. (B) Two pharmacologically distinct, prominently TrkB signaling activating agents (clozapine, CLZ; amitriptyline, AMI) induced a prominent decrease in locomotor activity over 30 min after administration, whereas an opposite effect was observed after AMPH. Control treatments and ATI had no significant effect on the locomotor activity of the animals. (C) Sedation induced by CLZ and AMI, as measured by decrease in locomotor activity, is reflected in the magnitude of TrkB phosphorylation over the control treatment, while treatments with either negligible (ATI and vehicle solution of CLZ) or stimulatory behavioral effects were not found to alter the signaling. Data in (A) are presented as mean ± standard error of mean (S.E.M.). In (B), the box and whiskers plot show the range of all data points, with the box representing the values between the 25 and 75 percentiles. A line within the box marks group median. * ≤ 0.05, ** ≤ 0.01, *** ≤ 0.001, **** ≤ 0.0001 (for statistical analyses and *n* numbers, see Supplementary Table S1). Abbreviations: GSK3β, glycogen synthase kinase 3β; p70S6K, ribosomal protein S6 kinase.

We began by testing several pharmacological substances for their effects on the phosphorylation of TrkB receptors and phosphorylation of selected downstream signaling pathways implicated in antidepressant action, including p70S6K (downstream of mTOR) and GSK3β.^1,3,35^ To this end, we administered mice drugs commonly classified as stimulants, antidepressants, sedatives, and volatile anesthetics, in dosages commonly utilized in the available literature. At 30 min after administration or continuous inhalation anesthesia, we collected samples from the medial prefrontal cortex (mPFC) for western blot (Figure 1A). We found that urethane (NMDA receptor/GABA_A_ receptor modulator), medetomidine (α_2_-adrenergic agonist), hydroxyzine (H_1_ receptor inverse agonist), isoflurane (volatile anesthetic), gamma-hydroxybutyrate (GHB; GABA_B_ receptor agonist), and antipsychotics such as clozapine (atypical antipsychotic) and chlorpromazine (typical antipsychotic) activated TrkB signaling (Figure 1A) and its downstream cascades (Figure S1, Supplementary File). Several drugs classified as antidepressants (amitriptyline, mianserin, and fluoxetine) also increased TrkB signaling or had a trend for increase (mirtazapine). However, antidepressants paroxetine and duloxetine, or stimulants such as amphetamine (dopaminergic stimulant) or atipamezole (α_2_-adrenergic antagonist) had no acute effect on TrkB signaling at the selected dose and time point. Notably, selective serotonin reuptake inhibitors (SSRIs) like fluoxetine are often considered nonsedative in the clinical context, but high acute doses commonly used in rodent antidepressant literature have shown to significantly reduce locomotor activity.^36^ Moreover, tricyclic antidepressants like amitriptyline are known to be sedatives.^37^ To screen for an association between sedation and TrkB phosphorylation, we tested selected compounds for their effects on locomotor activity (Figure 1B). Compared to vehicle-treated mice, clozapine and amitriptyline significantly reduced total ambulatory distance over 30 min, whereas an opposite effect was observed after amphetamine. Decreased locomotor activity was reflected in the magnitude of TrkB phosphorylation (Figure 1C). Altogether, these findings show that numerous pharmacological agents can activate TrkB signaling regardless of their clinical efficacy as an antidepressant and suggest that their propensity to induce behavioral sedation may be associated with the observed signaling (Figure 1C).

### Altered Bioenergetics, Sedation, and TrkB Signaling

**Figure 2:**
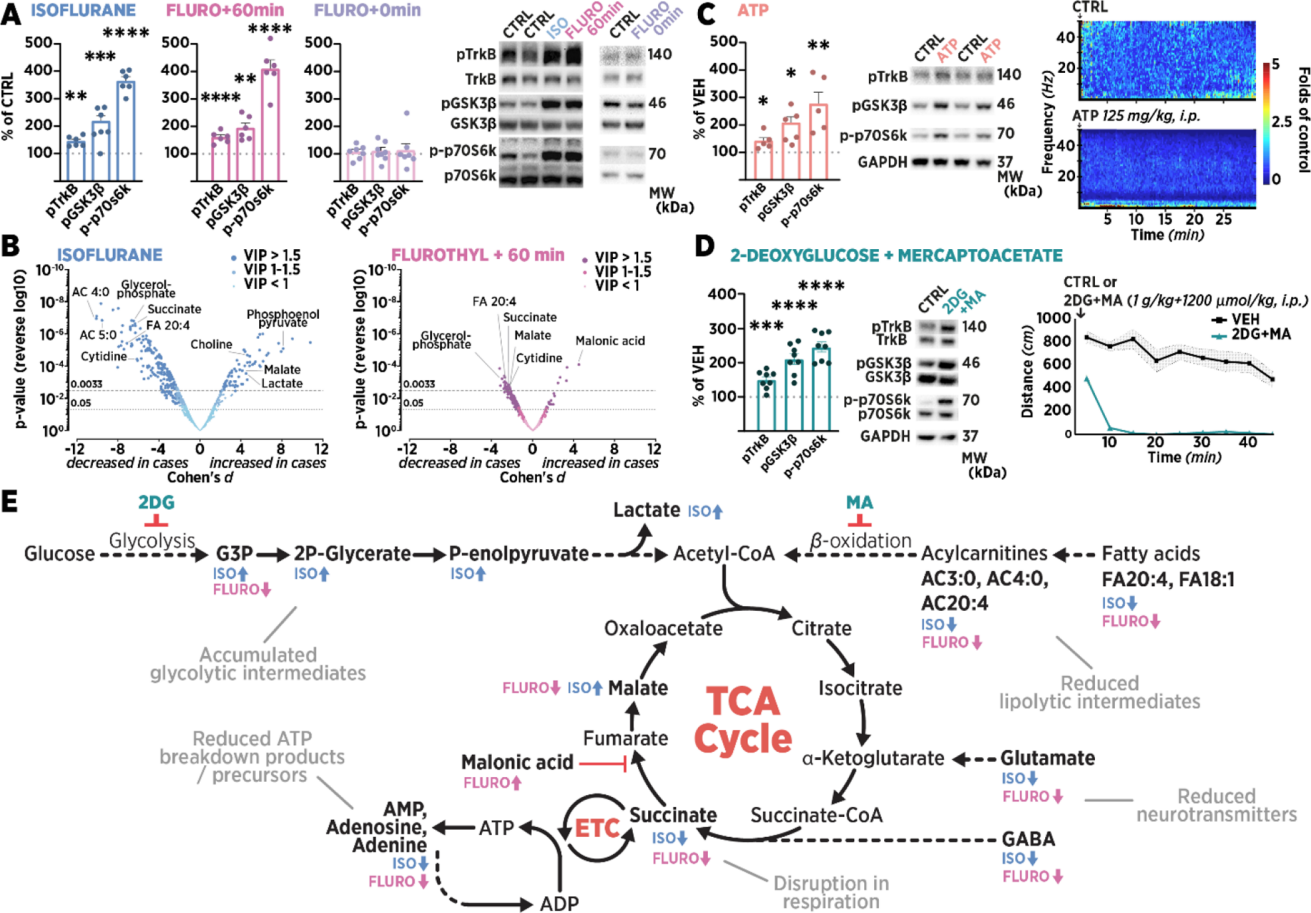
Metabolism and TrkB signaling. (A) TrkB^Y816^, GSK3β^S9^, and p70SK6^T421/S424^ phosphorylation levels remained regulated during 1 h isoflurane anesthesia and 1 h after a flurothyl-induced seizure. (B) Continuous isoflurane anesthesia for 1 h and 1 h recovery from brief exposure to flurothyl induce numerous changes in the metabolite profile of mouse prefrontal cortex. The volcano plot demonstrates that the effects of isoflurane are more pronounced than those of flurothyl. For detailed changes, see Supplementary File B. (C) Adenosine triphosphate (ATP; 125 mg/kg, intraperitoneal [i.p.]) produces a torpor-like state accompanied by electroencephalographic slowing and concomitant TrkB signaling. (D) Another model of torpor, inhibition of glycolysis, and lipid beta-oxidation by injection of 2-deoxy-d-glucose and mercaptoacetate (2DG and MA; 1 g/kg and 1200 μmol/kg, respectively, i.p.) induces sedation as assessed by behavioral immobility, and TrkB signaling. (E) Overview of the bioenergetic changes found in nontargeted metabolomics of samples corresponding to treatments of A and B. One hour of isoflurane anesthesia and 1 h recovery after flurothyl seizure both reduced succinate levels, which couples the tricarboxylic acid (TCA) cycle to the electron transport chain (ECT) to produce ATP. Both treatments also significantly decreased purinergic breakdown products of ATP, fatty acids (FA), and acylcarnitines (AC). Glycolytic intermediates were accumulated. Inhibition of primary metabolic pathways in vivo using 2DG and MA induces the same sedative phenotype and neurotrophic signaling as other antidepressant drugs. Phosphoproteins were normalized against the corresponding total protein signal and compared to the control group set to 100%. Data are presented as mean ± S.E.M. * ≤ 0.05, ** ≤ 0.01, *** ≤ 0.001, **** ≤ 0.0001 (for statistical analyses and *n* numbers, see Table S1). Abbreviations: ADP, adenosine diphosphate; AMP, adenosine monophosphate; CoA, coenzyme A; G3P, glyceraldehyde-3-phosphate; GSK3β, glycogen synthase kinase 3β; 2P-Glycerate, 2-phosphoglyceric acid; P-enolpyruvate, phosphoenolpyruvate; p70S6K, ribosomal protein S6 kinase.

To further understand the mechanism behind TrkB activation, we analyzed the molecular signaling events after treatment with two volatile drugs sharing a similar chemical structure but opposite pharmacological effect: flurothyl and isoflurane. Inhalation of flurothyl vapor over a period of few minutes evokes a generalized seizure by progressive excitation of the brain,^16^ whereas isoflurane dose-dependently suppresses neuronal activity, resulting in anesthesia.^25^ TrkB signaling was observed to be unaffected in brain samples collected immediately after the flurothyl-induced seizure, suggesting that the acute convulsive activity and the preceding excitation, during which there should conceivably be more activity-dependent release of BDNF, do not have an immediate effect on the phosphorylation levels of TrkB (Figure 2A). However, when the samples were collected an hour after the seizure (i.e., during the post-ictal state), significant TrkB signaling was observed, which was similar to the samples from animals subjected to continuous isoflurane anesthesia (Figure 2A). Our previous research has shown both states to be characterized by prominent sedation and suppression of cortical EEG activity.^16,25^ According to previous research, both anesthesia^38^ and recovery from excitatory stimuli^39,40^ are also characterized by hypothermia, suggesting that these states share some features at the face value. These findings prompted us to investigate the physiological correlates of recovery from a flurothyl seizure and ongoing anesthesia using a nontargeted metabolomics screen (Figure 2B,E, Supplementary File B). Overall, in comparison with recovery from a seizure, isoflurane anesthesia produced more prominent and often opposing changes in the metabolome. However, both treatments produced remarkably similar effects on bioenergetic targets involved in adenosine triphosphate (ATP) breakdown and synthesis, lipolysis, and neurotransmission. Moreover, both reduced the levels of succinate, which couples the tricyclic acid (TCA) cycle to the electron transport chain (ETC), signifying a deleterious effect on ATP production at 1 h of continuous volatile anesthesia or 1 h of recovery after seizure.^41^ Various sedative-anesthetic compounds have been previously shown to induce similar impairment of ATP production by mainly inhibiting cell respiration through ETC complexes I and II. The ETC complexes are readily inhibited by a range of pharmacological agents of distinct classification, most notably including conventional antidepressants^42,43^ and antipsychotics.^44^ The metabolism-inhibiting drugs vary considerably in their targets, working also through unspecified disruptive effects on mitochondrial lipid membrane dynamics (fluoxetine)^45^ or inhibiting complex IV^46^ and increasing energy-consuming cellular processes^47^ (ketamine). The metabolomic profile after flurothyl seizure, on the other hand, suggests that the decrease in the metabolic rate is a result of homeostatic inhibition of the complex II (succinate dehydrogenase) in response to the preceding excitatory insult. A similar inhibitory mechanism has been recently described during mammalian hibernation.^48^

Given the similarity in the metabolic effect induced by pharmacologically distinct isoflurane and flurothyl, we tested whether the pharmacological induction of the analogous metabolic state would result in activation of TrkB signaling. Two treatments common in the research of mammalian hibernation and torpor were used: ATP^49^ and a combination of metabolic inhibitors 2-deoxy-d-glucose and mercaptoacetate (2DG + MA).^50^ Systemic delivery of ATP slows down the TCA cycle through allosteric inhibition of pyruvate hydrogenase,^51^ producing a state characterized by immobility^52^ and increased slow EEG activity (∼1–4 Hz; Figure 2C). mPFC samples obtained 30 min after injection of ATP showed increased phosphorylation of TrkB^Y816^, GSK3β^S9^, and p70SK6^T421/S424^ (Figure 2C). Administration of 2DG + MA, which inhibit the first steps of both glycolysis (hexokinase) and lipid beta-oxidation (fatty-acid CoA dehydrogenase), respectively, also induced profound sedation and TrkB signaling (Figure 2D). An overview of the metabolomic changes produced by isoflurane and flurothyl, along with the targets affected by 2DG + MA, is presented in Figure 2E. Together, these findings suggest that antidepressant-associated TrkB signaling is activated by two seemingly opposing treatments that share the ability to disrupt the bioenergetic balance, or by specific inhibition of metabolic pathways upstream of the TCA cycle, leading to a sedative phenotype and activation of antidepressant-associated neurotrophic signaling.

### TrkB Phosphorylation Is Not Causally Connected to SWA

**Figure 3:**
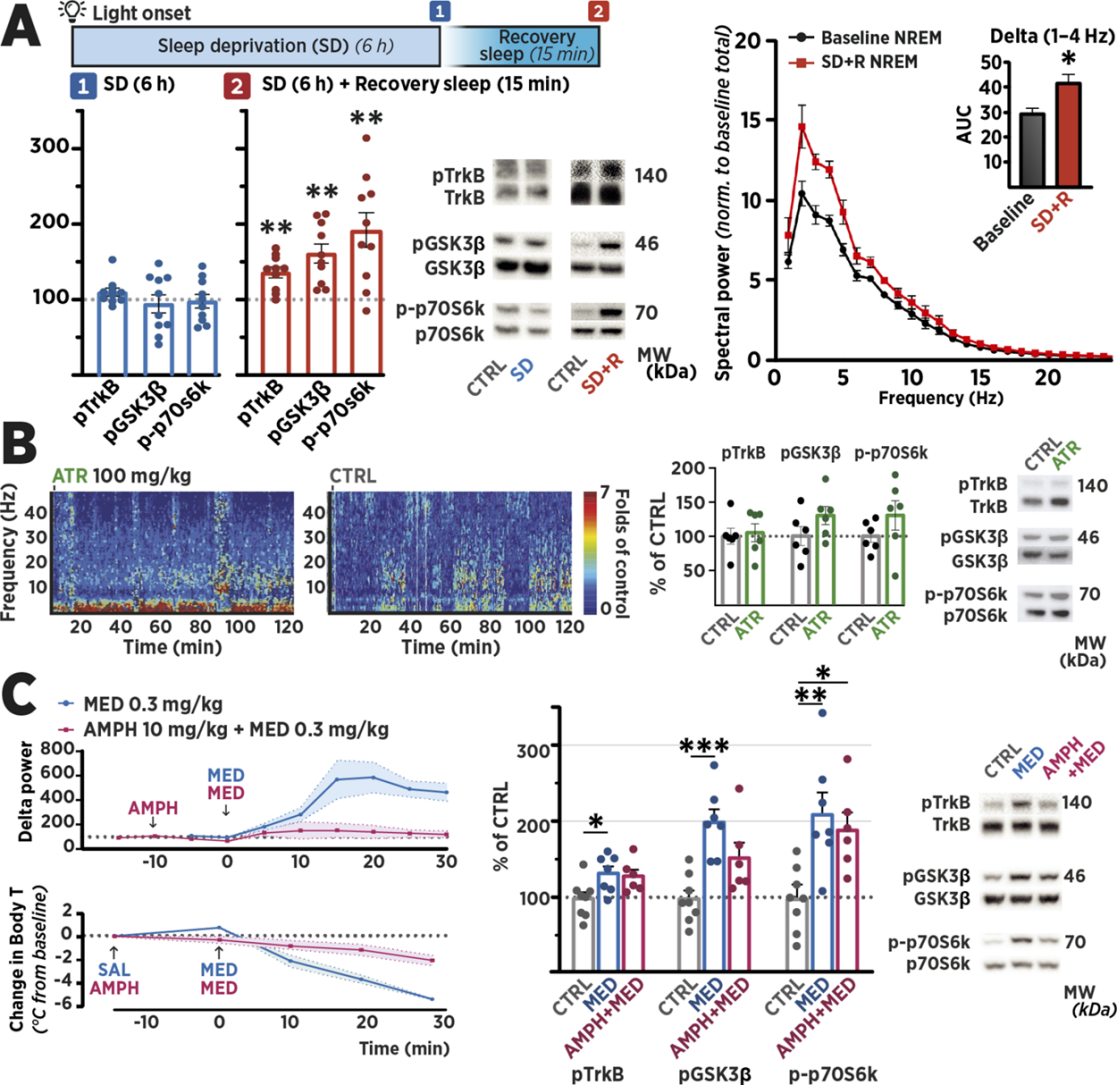
Sleep, cortical slow-wave activity, and phosphorylation of TrkB and associated targets. (A) Slow-wave activity within the delta band (1–4 Hz) is increased significantly during nonrapid eye movement (NREM) sleep of the 1.5 h recovery sleep (SD + R) following 6 h sleep deprivation (SD). Baseline NREM represents the spectral power of NREM sleep over 24 h before SD. For western blot, medial prefrontal cortex samples were collected immediately after 6 h of SD or 15 min after the animals entered recovery sleep based on their immobility and posture. Phosphorylation of TrkB^Y816^, GSK3β^S9^, and p70SK6^T421/S424^ remained unaltered at the end of SD, whereas a statistically significant increase in TrkB^Y816^, GSK3β^S9^, and p70SK6^T421/S424^ is observed in the samples obtained from animals allowed to enter recovery sleep (SD + R). Representative blot images with corresponding molecular weights (MW) are shown. Phosphoproteins were normalized against the total protein signal and compared to the control group set to 100%. Data are presented as mean ± standard error of mean. * ≤ 0.05 (for statistical analyses and *n* numbers, see Table S1). Abbreviations: GSK3β, glycogen synthase kinase 3β; p70S6K, ribosomal protein S6 kinase; SD-R, sleep deprivation + recovery. (B) EEG recorded from a prefrontal cortex-implanted electrode for 2 h after atropine (ATR; 100 mg/kg, i.p.) injection shows marked reduction in electrophysiological activity, while a negligible effect on animal phenotype was observed. EEG of vehicle-treated animals (CTRL) shown on right. Medial prefrontal cortex samples collected from atropine-treated animals 30 min after injection show insignificant change in the of TrkB^Y816^, GSK3β^S9^, and p70SK6^T421/S424^. (C) Impact of amphetamine (AMPH; 10 mg/kg, i.p.) pretreatment on medetomidine (MED; 0.3 mg/kg, i.p.)-induced SWA and decrease in core body temperature (Body T). AMPH did not significantly affect MED-induced activation of TrkB signaling in the medial prefrontal cortex, while the increase in SWA was abolished. Phosphoproteins were normalized against the total protein signal and compared to the control group set to 100%. Data are presented as mean ± S.E.M. * ≤ 0.05, ** ≤ 0.01, *** ≤ 0.001 (for statistical analyses and *n* numbers, see Table S1).

To investigate the role of endogenous homeostatic and physiological mechanisms in the activation of TrkB, we decided to use SD, a nonpharmacological and clinically effective treatment of depression,^21^ to evaluate the possible association between TrkB signaling and SWA. Throughout wakefulness, there is a progressive increase in neuronal excitability and energy demand, which accumulate sleep pressure.^53^ When the extended wakefulness of SD ceases, the subject enters deep recovery sleep, which is characterized by increased SWA that declines over the bout of sleep as the sleep pressure is relieved.^54^ Thus, at the first glance, the course of the intervention shares some characteristics with the brief flurothyl exposure discussed above—a period of excitation followed by a period of slowing down EEG activity. To test whether a similar increase in TrkB signaling occurs during SD-induced recovery sleep, we subjected animals to 6 h of SD starting at the beginning of the light period (Zeitgeber time 0 [ZT0]), after which a subset of animals was allowed to enter recovery sleep in their home cage, monitored remotely for behavioral immobility and a sleeping posture. As expected, recovery sleep after SD increased the spectral power of the slowest EEG bands, most notably in the SWA range (∼1–4 Hz; Figure 3A). Brain samples from animals terminated at the end of the SD period demonstrated a negligible effect on phosphorylation of TrkB^Y816^, GSK3β^S9^, and p70SK6^T421/S424^ in comparison to control. However, analogous to the delayed onset of signaling observed after flurothyl seizure, increased phosphorylation of the signaling pathway was observed in the samples collected from mice that were allowed to enter recovery sleep for 15 min before sample collection (Figure 3A).

To establish whether the reduced electrophysiological activity plays a causal role in the activation of TrkB signaling, we utilized the muscarinic acetylcholine receptor antagonist atropine. Under the influence of atropine, pronounced cortical SWA is observed in electrophysiological recordings, yet the behavior of rodents remains relatively normal, and their subcortical regions remain aroused.^55^ To test whether the TrkB signaling is activated in the cortex but unaffected in lower regions, mPFC and midbrain samples were collected 30 min after atropine injection. Despite robust increase of cortical EEG SWA in atropine-treated mice, phosphorylation levels of TrkB^Y816^, GSK3β^S9^, and p70SK6^T421/S424^ remained unaltered in both the mPFC and midbrain samples (Figures 3B and S2). This suggests that the mere presence of cortical SWA is not sufficient to explain the observed TrkB phosphorylation, and we continued to investigate whether the sedation-activated TrkB signaling could be prevented by reversing other physiological changes associated with deep sleep or sedation. To this end, we used medetomidine, an alpha-2 receptor agonist, due to its ability to induce a sleeplike state coinciding with SWA and TrkB signaling,^16,56^ and amphetamine, which is known to augment EEG activity,^57^ wakefulness,^58^ and thermogenesis.^59^ We replicated the previously observed medetomidine-induced upregulation of TrkB^Y816^, GSK3β^S9^, and p70SK6^T421/S424^ in samples collected from the PFC and hippocampus.^16^ Furthermore, we detected comparable signaling changes in both the synaptosome preparations and total homogenates of the samples, suggesting that the observed response is not isolated to the synaptic fraction (Figure S3). Pre-treatment with amphetamine abolished the medetomidine-induced increase in EEG delta power but did not significantly affect TrkB phosphorylation in the mPFC (Figure 3C) or cerebellum samples (Figure S4A), which were collected to illustrate the occurrence of sedation-induced TrkB signaling throughout the brain in different structures. Together, these results further support the hypothesis that SWA is not causally connected with TrkB phosphorylation. However, body temperature measurements recorded during the experiment suggested that while the effect on EEG was abolished with amphetamine pre-treatment, it had only a small effect on the medetomidine-induced decrease in the rectally measured core body temperature (Figure 3C). Since the effects of amphetamine on medetomidine-induced changes in SWA and TrkB signaling could be explained pharmacologically through their opposing effects on noradrenergic neurotransmission, we next tested whether the signaling can be regulated directly through temperature.

### Modulation of TrkB Activation through Thermoregulation

Homeothermic animals maintain a relatively constant body temperature in a wide range of ambient temperatures by altering their metabolic rate. Consequently, the cerebral metabolic rate, for example, changes linearly with the surrounding temperature and energy-consuming functional activity of the neurons.^60−62^ Due to the observed effect of amphetamine and medetomidine on the body temperature, we set to study whether the drug-induced TrkB signaling can be manipulated using exogenously applied heat during the acute effects. To this end, a cohort of mice was injected with medetomidine and placed in cages located either in the standard laboratory room temperature (*T*_A_ = 22 ± 1 °C) or an incubator maintained at an elevated temperature (37 ± 1 °C). Core body temperature was measured rectally at 10 min intervals, and the mice were terminated for biochemical analyses at 30 min after administration. Again, medetomidine induced a progressive reduction in body temperature of the animals at room temperature, which was not seen in the heated animals (Figure S4B). Remarkably, prevention of the medetomidine-induced decrease in body temperature also reduced the phosphorylation of TrkB^Y816^, GSK3β^S9^, and p70SK6^T421/S424^ (Figure S4C).

**Figure 4:**
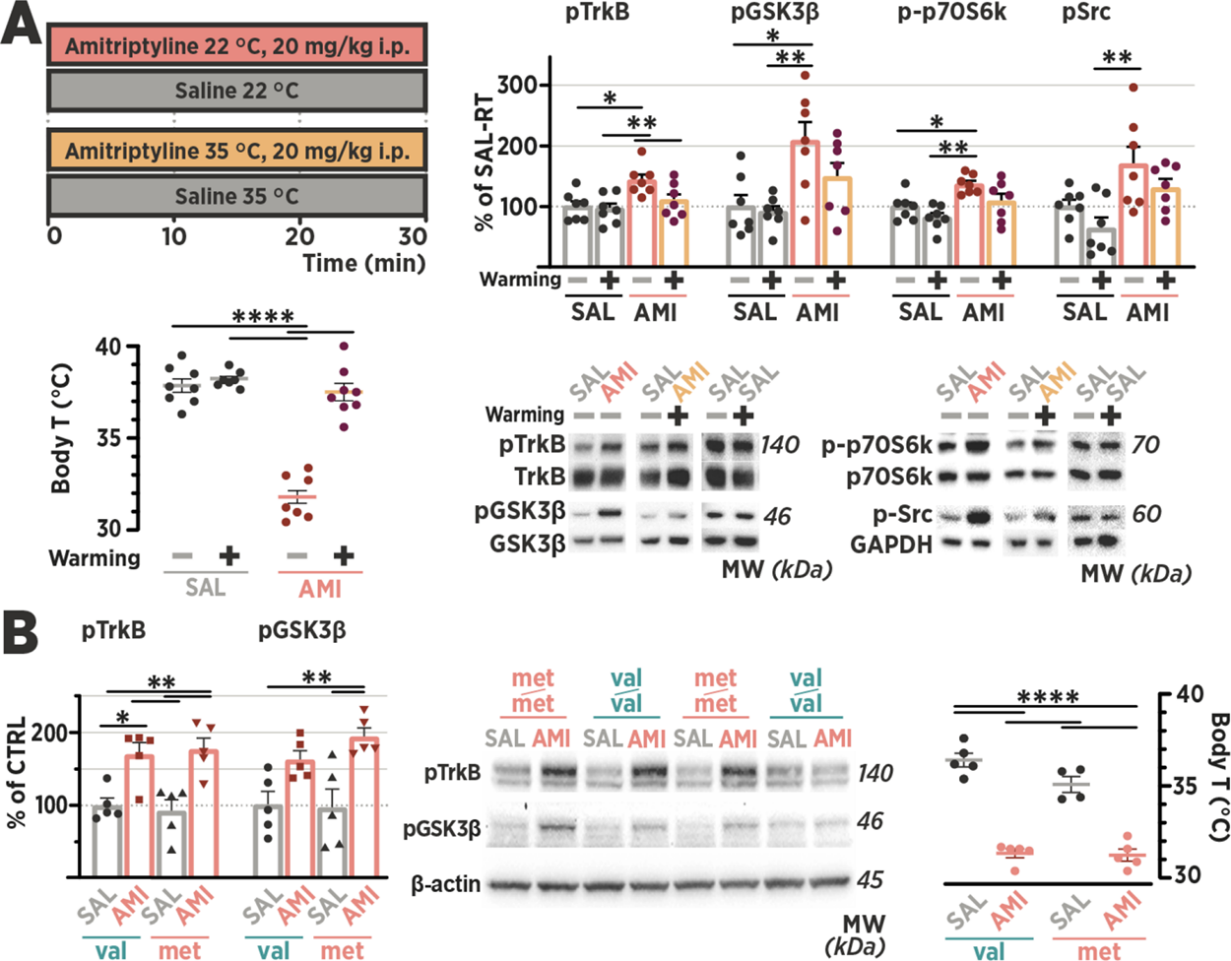
Antidepressants transactivate TrkB through a BDNF-independent, temperature-dependent mechanism. (A) By 30 min after injection, amitriptyline (AMI; 20 mg/kg, intraperitoneal) caused a significant decrease in the body temperature (Body T) of animals housed at ambient room temperature (T_A_ 22 ± 1 °C). Housing the animals in a warm incubator (35 ± 1 °C), which had negligible effects on the Body T of saline-treated animals (SAL), blunts the hypothermic effect of AMI and inhibited the AMI-induced phosphorylation of TrkB^Y816^, in the medial prefrontal cortex samples collected at 30 min after injection. (B) AMI readily activates TrkB^Y816^ and GSK3β^S9^ phosphorylation in the medial prefrontal cortex, along with hypothermia, in both Bdnf^val66val^ and Bdnf^met66met^ mice. Phosphoproteins were normalized against corresponding total protein and compared to the control group set to 100%. Data are presented as mean ± S.E.M. * ≤ 0.05, ** ≤ 0.01, *** ≤ 0.001, **** ≤ 0.0001 (for statistical analyses and *n* numbers see Table S1). Abbreviations: BDNF, brain-derived neurotrophic factor; GSK3β, glycogen synthase kinase 3β.

Next, we set to investigate the role of temperature and metabolic rate in the antidepressant-induced TrkB signaling. We decided to use the tricyclic antidepressant amitriptyline, as it has been claimed to be—among many other drugs—a direct agonist of the TrkB receptor.^8^ We studied the acute effects of amitriptyline using a dose (20 mg/kg, i.p.) used in previous preclinical antidepressant research.^63,64^ The treatment produced marked sedation and hypothermia in mice at room temperature, accompanied by phosphorylation of TrkB^Y816^, GSK3β^S9^, and p70SK6^T421/S424^ in the mPFC samples (Figure 4A). Akin to the observation seen with medetomidine, elevation of ambient temperature abolished the effect of amitriptyline on both the body temperature and TrkB signaling (Figure 4A), with a negligible effect on the hyperglycemic response induced by amitriptyline (Figure S5). Thus, we screened selected drugs and treatments that have previously demonstrated robust TrkB activation, including 2DG + MA, chlorpromazine, isoflurane, and flurothyl, for their effects on the body temperature and found that they all induced prominent decrease in either rectal or skin temperature when administered to animals housed at room temperature (Figure S6). We also repeated the experiment with isoflurane anesthesia in a temperature-controlled setting and observed a similar pattern of abolished TrkB phosphorylation when the body temperature was maintained (Figure S4D). These observations question the widely accepted claims, according to which the effects of amitriptyline and various other antidepressants or sedative-anesthetic drugs are mediated by direct pharmacodynamic action with the TrkB receptor, suggesting instead a causal effect from their ability to impair energy metabolism, and consequently, lower body temperature.

As mentioned previously, some of the mechanisms that have been proposed for antidepressant-induced TrkB activation involve enhanced BDNF expression, translation, and release. We have previously shown that both imipramine and isoflurane are able to activate the brain TrkB signaling in BDNF-deficient mice,^12,24^ alluding instead to a BDNF-independent transactivation mechanism. To test this notion further, we administered an acute dose of amitriptyline to adult mice carrying Met66Met mutation in the *Bdnf* gene, which impairs activity-dependent BDNF release.^65,66^ The magnitude of the TrkB signaling and thermoregulatory responses induced by amitriptyline were indistinguishable between the *Bdnf^met66met^* and control mice (*Bdnf^val66val^*) (Figure 4B).

Finally, considering the role of Src family kinases in the neurotrophin-independent Trk receptor transactivation,^67^ as well as mediating temperature-associated changes in the metabolic rate,^68^ we assessed the active phosphorylation of Src in brain homogenates that showed increased TrkB phosphorylation. Indeed, Src^Y416^ phosphorylation was rapidly induced by amitriptyline, isoflurane, metabolic inhibitors 2-DG + MA, and during recovery from SD and flurothyl seizure (Figures 4A and S7). Conversely, the kinase phosphorylation in medetomidine- and amitriptyline-treated animals was reduced by warm ambient temperature, although the change was not statistically significant with amitriptyline (Figure 4A). A noncanonical pathway of TrkB activation in the described phenomenon is further supported by the lack of concomitant phosphorylation of extracellular-signal regulated kinase (ERK1/2; Figure S8), which is a well-established downstream target of BDNF-induced TrkB activation.^69^

## Discussion

The ability of antidepressants to acutely activate neurotrophic factor receptor TrkB in rodents was established in the seminal observations by Saarelainen et al.,^5^ yet the underlying mechanisms have remained enigmatic. Attempts to explain the effects of antidepressants on TrkB signaling have revolved around canonical principles of receptor pharmacology, with some studies suggesting that all antidepressants, including structurally different amitriptyline,^8,9^ fluoxetine,^13^ and ketamine,^13^ directly bind to the receptor. Rodent studies have also indicated that antidepressant-like effects and their associated molecular and structural changes are coupled to TrkB downstream signaling pathways, most notably the activation of mTOR and its effector P70S6K and the inhibition of GSK3β.^1,3,35^ In this study, we show that the phosphorylation of TrkB, P70S6K, and GSK3β is regulated in mice by various classical antidepressants, as well as numerous sedative-anesthetic drugs lacking any established clinical value in the treatment of depression. Moreover, we demonstrate that the activation of neurotrophic signaling cascade occurs independently of any direct pharmacodynamic action during recovery from a convulsion or in physiological sleep following sleep deprivation.

As suggested by the four key findings of this report, the TrkB-activating interventions appear to be connected by their capability of producing sedation and decreasing metabolic rate. First, a range of pharmacodynamically distinct sedative agents were found to activate TrkB irrespective of the drugs known for the clinical antidepressant effect. Second, flurothyl and isoflurane, despite their distinct mechanism of action and timeframe of activating TrkB, led to a similar metabolomic profile that suggested perturbed bioenergetics. Third, activation of TrkB signaling was observed during a sedative-hypothermic state induced by specific metabolic inhibitors, which are routinely used to model hypothermia. Fourth, the activation of TrkB can be prevented by controlling the ambient temperature to reverse the drug-induced hypothermia. This novel hypothesis of a noncanonical drug-induced activation mechanism is reinforced by our results, showing that the antidepressant-induced TrkB signaling is not compromised in animals with impaired activity-dependent BDNF release but instead can be readily prevented through the manipulation of ambient temperature, conceivably by its effect on body temperature, which is determined by the metabolic activity.^70,71^

From a pharmacological perspective, drugs that produced consistent TrkB activation belonged to the general categories of either sedative-anesthetics, metabolic inhibitors, or antidepressants and antipsychotics that have sedating properties at high doses. Out of drugs categorized as antidepressants, fluoxetine, mirtazapine, amitriptyline, and mianserin increased TrkB, p70S6K, and GSK3β phosphorylation, while duloxetine and paroxetine did not. Mirtazapine, amitriptyline, and mianserin are generally considered to have pronounced sedative properties and are sometimes used to treat insomnia, while the SSRIs fluoxetine and paroxetine, and the selective serotonin and noradrenaline reuptake inhibitor (SNRI) duloxetine, are generally considered to be nonsedative. However, high acute doses of fluoxetine have been shown to significantly reduce body temperature^72^ and locomotor activity in rodents,^36^ conceivably reflecting its effect on bioenergetics. Small laboratory rodents spend a considerably higher proportion of their basal metabolic rate to maintain their body temperature, in comparison to humans; thus, clinically insignificant perturbations to central and peripheral thermoregulatory mechanisms can have a considerably different effect in preclinical studies,^73−75^ which are conducted at an ambient temperature far below the animals’ thermoneutrality. Since our screening was limited to a single dose of each drug and was designed to address TrkB phosphorylation, rather than sedation and other complex interactions of physiology and environment, subsequent dose–response studies in a controlled experimental setting are warranted. Nevertheless, we found that the sedation induced by clozapine and amitriptyline, as measured by a decrease in locomotor activity, was reflected in increased TrkB phosphorylation, whereas stimulants amphetamine and atipamezole did not alter the signaling. Similarly, increased TrkB signaling and decreased locomotor activity and body temperature were observed after the administration of metabolic inhibitors 2DG and MA. These findings are in line with previous studies associating TrkB activation induced by medetomidine, nitrous oxide, and ketamine with increased SWA in mice.^16,26^ Strikingly, we have not observed acute activation of TrkB signaling using subanesthetic doses of ketamine, commonly used in antidepressant research, but instead with anesthetic doses that have been shown to elicit hypothermia.^76,77^

While the functional role of the observed phenomenon remains unclear, our findings implicate that TrkB activation occurs during natural deep slow-wave sleep, known to coincide with decreasing body temperature, as evidenced by our experiment using sleep deprivation. By using amphetamine to block medetomidine-induced increase in SWA and atropine to induce robust cortical SWA, we demonstrated that SWA is not, however, in and of itself a sufficient causal explanation for the increased signaling. Instead, TrkB signaling induced by the antidepressant amitriptyline and sedative-anesthetics medetomidine and isoflurane was specifically abolished by the active maintenance of body temperature. Given that the depth of sedation is dependent on ambient temperature and NREM sleep is accompanied by brain cooling,^78^ further research on the thermal dynamics of TrkB signaling and sleep could perchance shed light on the putative physiological role of temperature regulation in neuroplasticity and possibly in producing antidepressant effects. Notably, recent studies have shown that brief therapeutic hyperthermia can elicit antidepressant effects in depressed patients,^79^ which have been associated with the subsequent facilitation of thermoregulatory cooling.^80^ Temperature-induced antidepressant-like effects have also been observed in rodents.^81^ As a limitation of this study, we used naïve mice and focused solely on characterizing the temperature-dependent signaling phenomena. Future studies investigating the functional significance of the observed phenomena in animal models of depression, and in producing antidepressant-like behavioral outcomes, are warranted.

The findings of this study may also provide further insight into the neurobiological mechanisms of therapeutic hypothermia. Hypothermia, among the oldest known neuroprotective treatments,^82^ has been shown to rescue the brain from various insults and is associated with significant physiological structural plasticity.^83,84^ The therapeutic effects of hypothermia are widely considered to be mediated through attenuated energy expenditure, oxidative stress, and inflammation, with recent studies also highlighting the involvement of TrkB signaling. In particular, Peretti and colleagues have shown that RNA-binding motif 3 (RBM3)—a major inducer of cold-induced structural plasticity and neuroprotection^85^—controls the phospholipase-Cγ signaling downstream of TrkB.^86^ The authors used a combination of pharmacological (adenosine monophosphate [5′-AMP]) and environmental (low ambient temperature) means to induce and maintain hypothermia, respectively, observing upregulated BDNF release and TrkB signaling both in vivo and in vitro. In contrast to the considerably longer, up to 24 h-long time frames used in the previous experiments, our findings demonstrate that even an acute decrease in body temperature can upregulate TrkB signaling within minutes.

This report contradicts with previous hypotheses, according to which the acute effects of antidepressants on TrkB signaling are mediated by a direct receptor-ligand interaction, or via BDNF release, due to two major reasons. (1) The effect of amitriptyline on TrkB signaling is abolished if the hypothermic reaction is prevented by adjusting ambient temperature. (2) There is an identical signaling response seen in animals with genetically abated BDNF release or function.^12,87^ Furthermore, we observed a concomitant reduction—rather than an increase—of ERK phosphorylation, distinguishing the current phenomenon from canonical BDNF-mediated activation of TrkB.^69,88,89^ This report therefore extends our previous observations that support the hypothesis of BDNF-independent activation of TrkB by antidepressants.^12,24^ While the precise mechanism of the activation remains to be fully elucidated, one possibility is the involvement of Src family kinases, which have been previously associated with transactivation of the TrkB.^67^ Previous studies have shown that Src, as well as several other targets associated with TrkB signaling (most notably GSK3β), are indeed regulated by the metabolic milieu through mechanisms such as oxidative stress and altered neuronal lipid environment.^90,91^

Beyond the implications to therapeutic mechanisms of various drugs, the findings of this report raise crucial questions regarding the reproducibility and translational value of the neuropharmacological research. Over a third of the energy expenditure of a mouse goes to maintenance of body temperature at a standard laboratory *T*_A_ of 22 °C.^92^ Thus, the mice grown and experimented with in a constant cold environment differ in their phenotype, metabolism, and thermal responses from those raised, akin to humans, closer to their thermoneutral zone (∼30 °C).^93^ Further variability is introduced by the nonstandardized timing of experiments, as the thermoneutrality of laboratory rodents undergoes circadian variation. Thus, it is conceivable that the available literature on TrkB signaling is significantly affected by varying conditions related to animal housing group sizes, thermal, and air circulation conditions.^94^ Moreover, previous studies have demonstrated that chronic stress, used to study depression-like behavior in rodents, modulates thermoregulatory cooling mechanisms,^95^ which may further influence drug effects. Currently, the reporting of these fundamental experimental factors is lacking in the pharmacological literature, and data regarding the thermal responses produced by common psychopharmacological agents are very limited.

In conclusion, by studying various antidepressants and sedative-anesthetic drugs, we found that their propensity to activate TrkB receptor signaling is coupled to their ability to alter the thermal physiology in mice. Maintaining the animals at elevated temperatures during acute drug effects was found to completely abolish the signaling previously associated with the drugs’ pharmacological action. Moreover, our preliminary findings suggest that analogous TrkB activation is observed during physiological sleep, uncoupling the effect from solely a pharmacological domain. Most importantly, this report proposes a new hypothesis for acute antidepressant-induced TrkB activation by highlighting the role of bioenergetics and thermoregulation in the modulation of neurochemical signaling. This notion raises critical questions about the mechanistic aspects of TrkB signaling and calls for more carefully controlled experimental settings to re-evaluate the established understanding of drug-induced TrkB signaling by antidepressants and other treatments. Further study of these physiological processes may uncover novel ways to utilize such physiological adaptations in the treatment of neuropsychiatric conditions and recovery from brain insults.

## Methods

### Animals

Unless otherwise stated, 10–16-week-old C57BL/6JRccHsd mice (Envigo, Venray, Netherlands) were used. Animals were maintained under standard housing conditions (22 ± 1 °C, 12 h light–dark cycle, lights on at 6 A.M. or 9 A.M., humidity 40 ± 10%) in filtered plastic cages, with wood shavings and access to food and water available *ad libitum*. BDNF Val66Met mice were generated as previously described.^66^ Briefly, BDNF Val/Met mice were interbred, and the offspring were genotyped by polymerase chain reaction (PCR) analysis of tail tip-derived genomic DNA. The experiment involving BDNF Val66Met mice was conducted in female BDNF^Val/Val^ and BDNF^Met/Met^ mice. The animal experiments were carried out in compliance with the European Communities Council Directive of 22 September 2010 (Directive 2010/63/EU), according to the guidelines of the Society for Neuroscience and approved by the County Administrative Board of Southern Finland (Licenses ESAVI/9793/04.10.07/2016 and ESAVI/5844/2019).

### Drug Administrations

The following drugs were diluted in isotonic saline and administered intraperitoneally (i.p.) in a volume of 10 mL/kg: 2-deoxy-d-glucose (2-DG; 1 g/kg, i.p.; Sigma-Aldrich), adenosine triphosphate (ATP; 125 mg/kg, i.p., Sigma-Aldrich), amitriptyline-HCl (20 mg/kg; s.c.; Tocris), (*S*)-amphetamine-HCl (10 mg/kg; i.p.; Toronto Research Chemicals; kindly provided by Dr. Esa Korpi, University of Helsinki), atipamezole-HCl (1 mg/kg; s.c.; Antisedan, Orion Pharma), atropine sulfate (100 mg/kg, i.p., Sigma-Aldrich) chlorpromazine-HCl (10 mg/kg, i.p.; Orion Pharma), (*S*)-duloxetine-HCl (10 mg/kg; s.c.; Tocris), fluoxetine-HCl (20 mg/kg, i.p.; Bosche Scientific), gamma-hydroxybutyrate sodium (GHB; 275 mg/kg; i.p.; Xyrem; UCB Pharma Ltd.), hydroxyzine-HCl (50 mg/kg, s.c., Sigma-Aldrich), medetomidine-HCl (0.3 mg/kg; i.p.; Domitor; Orion Pharma), mercaptoacetate (MA; 1200 μmol/kg, i.p.; Sigma-Aldrich), mianserin-HCl (20 mg/kg; s.c.; Tocris), mirtazapine-HCl (20 mg/kg; s.c.; Tocris), paroxetine-HCl (10 mg/kg; s.c.; Sigma Aldrich), and urethane (2 g/kg; kindly provided by Dr. Kai Kaila, University of Helsinki). Clozapine (6 mg/kg, 10 mL/kg, i.p.; Sigma-Aldrich) was dissolved in 0.1 M hydrochloric acid, adjusted to pH ∼7 with 0.1 M sodium hydroxide, and then diluted to a concentration of 1.2 mg/mL with sterile saline. An equivalent vehicle solution was prepared without the active drug for clozapine control animals.

Isoflurane, sevoflurane, and flurothyl (bis(2,2,2-trifluoroethyl) ether; Sigma-Aldrich) were administered as previously described.^16,24^ Briefly, isoflurane anesthesia was induced using 2% isoflurane (Vetflurane, Virbac) and maintained under constant flow of 1.5% isoflurane.^24^ Sevoflurane anesthesia was induced using 6% sevoflurane (Sevoflurane Baxter 100%, Baxter Healthcare) and maintained under 4.5%.^96^ Volatile anesthetics were administered in an induction chamber of a small-animal anesthesia system (EZ-B800; World Precision Instruments, FL, USA) containing a preheated pad to prevent hypothermia. Sham-treated control animals received pressurized room air. Seizure was induced by injecting 10% flurothyl (in 90% ethanol) at a steady rate into a cotton pad placed inside the lid of an airtight Plexiglass chamber (13 × 13 × 13 cm) until the animals went into a myoclonic seizure. The lid was removed to terminate the seizure, after which the animals were moved to recover in their home cages. For control animals, 90% ethanol without flurothyl was injected to the pad as a sham treatment. All treatments were administered 3–7 h after the light onset (6 A.M.) in standard laboratory conditions, with animals brought to the room to habituate for at least 30 min before experiments.

### Locomotor Activity Recordings

The locomotor activity of a group of animals was recorded after administration of amitriptyline–HCl, amphetamine–HCl, clozapine, or atipamezole–HCl. Control animals were injected with isotonic sterile saline or clozapine vehicle solution. The treatments were selected from the array of pharmacological agents previously tested for their effects on TrkB signaling (Figure 1), and doses, routes of administration, and drug solution preparations were equivalent to those used in Figure 1A. Different treatments were administered in a randomized order throughout several sessions, separated by a washout period over 3–4 days. Animals were habituated, prior to drug administration, to the recording arena for 30 min in the first trial and 15 min in subsequent sessions. The locomotor activity was recorded for 30 min using the GeoVision Multicam system (GeoVision Inc., Taipei, Taiwan) and analyzed with EthoVision XT (Version 11.0.; Noldus Information Technology, Wageningen, the Netherlands).

### EEG Recordings

EEG-recordings were performed as described.^16^ Briefly, electrodes were implanted under isoflurane anesthesia using lidocaine (10 mg/mL) for local anesthesia and buprenorphine (0.1 mg/kg, s.c.) and carprofen (5 mg/kg, s.c.) for postoperative pain care. Two epidural screw EEG electrodes were placed above the fronto-parietal cortex. A third screw was installed as the mounting support. Two silver wire electrodes were implanted in the nuchal muscles to monitor electromyographic activity (EMG). After a recovery period (5–7 days), animals were connected to flexible counterbalanced cables for EEG/EMG recording and habituated to recording cables for three days. Recordings were conducted in the home-cages of the animals. The EEG and EMG signals were amplified (gain 5/10 K) and filtered (high pass: 0.3 Hz; low pass 100 Hz; notch filter) with a 16-channel AC amplifier (Model 3500, A-M Systems), sampled at 254 Hz or 70 Hz with 1401 interface (Cambridge Electronic Design Limited, Cambridge, U.K.), and recorded and processed using Spike2 software (Version 8.07, Cambridge Electronic Design Limited). EEG power spectra were calculated within the 1–50 Hz frequency range by fast Fourier transform (FFT = 256, Hanning window, 1.0 Hz resolution). Oscillation power in each bandwidth (delta = 1–4 Hz; theta = 4–7 Hz; alpha = 7–12 Hz; beta = 12–25 Hz; gamma low = 25–40 Hz; gamma high = 60–100 Hz) was computed in 30–300-second epochs from spectrograms (FFT size = 1024 points) for each animal. Representative sonograms were computed using a Hanning window with a block size of 512.

### Sleep Deprivation

Animals were kept awake in their home cages at standard housing conditions for 6 h starting from light onset (9 A.M.) by introducing novel objects or gentle handling whenever the animals began to display signs of sleepiness. Animals for biochemical analyses were euthanized using rapid cervical dislocation either immediately following the sleep deprivation or after 15 min of recovery sleep. The entry to recovery sleep was determined for each animal individually using a remote infrared camera based on their immobility and crouched posture typical for sleep. For EEG studies of SD, the power spectrum of the wake baseline was continuously recorded for 24 h to establish a baseline, followed by a 6 h SD and 1.5 h long recovery sleep period.

### Temperature Measurements and Thermoregulation

Core body temperature was measured using small-animal rectal probe thermometers (FHC Frederick Haer & Co, Bowdoin, ME, USA; 7001H). The temperature measurements, lasting less than 10 s, were performed by experienced personnel in order to minimize the impact of handling stress on body temperature. For longitudinal analysis of body temperature, whole-body thermal imaging was used to assess the surface temperature of the animals. Thermal images were acquired using a forward-looking infrared (FLIR) P640 thermal camera (FLIR Systems, Inc., Wilsonville, OR, United States; via Infradex Oy, Vantaa, Finland), with a reported thermal sensitivity of 0.06 °C at 30 ° C and a reading accuracy of ±2%. The camera was equipped with a 45° field of view, 19 mm focal length lens, and positioned perpendicular to the imaged plane above the recording chambers. Thermal data were acquired at a rate of one image per 30 s, for 3–5 min before and following times after treatment initiation; 2-DG + MA receiving animals were recorded for 50 min after injection; animals recovering from flurothyl-induced seizure were recorded for 45 min after the end of convulsing; and animals subjected to 1 and 20 min of isoflurane anesthesia were recorded for 7 and 10 min, respectively. Images were analyzed using either FLIR Tools software (version 6.4.18039.1003), where the highest radiometric pixel temperature was manually acquired for each rodent cage/image using the rectangle tool or ThermaCAM Researcher Pro (version 2.10), where the rectangle tool was used to automatically acquire the highest pixel temperatures for each rodent cage/image.

In the experiments involving controlled ambient temperature, animals were heated using a ventilated incubator (Vet-Tech Solutions Ltd., Congleton, United Kingdom) set to maintain the temperature of the chamber at the upper limit of animals’ thermoneutral zone (35 °C)^75^ to account for sedation-associated hypothermia. An external thermometer placed on the incubator floor was used to confirm the chamber temperature, recording *T*_A_ range of 33–34 °C during the experiments. Control animals were placed in empty plastic containers of roughly the same size as the incubator, kept at *T*_A_ of 22 °C. The temporary conditions did not include other mice or wood shavings affecting the microenvironment of the animals. Core temperature measurements at the time of termination were acquired using rectal probes as described above.

### Dissection and Processing of Brain Samples

Animals were euthanized at indicated times by a rapid cervical dislocation followed by decapitation and removal of the brain. Bilateral mPFC (including prelimbic and infralimbic cortices) was rapidly dissected on a cooled dish and stored at −80 °C until further processing. For some experiments, hippocampal (HC), cerebellar (CB), and/or midbrain (MB) samples were collected and processed with the same protocol. For the analysis of crude brain homogenates, the samples were homogenized in lysis buffer (137 mM NaCl, 20 mM Tris, 1% NP-40, 10% glycerol, 48 mM NaF, H_2_O, Pierce Protease Inhibitor Mini Tablet [Thermo Scientific; Waltham, MA, USA], Pierce Phosphatase Inhibitor Mini Tablet [Thermo Scientific; Waltham, MA, USA]). After ∼15 min incubation on ice, samples were centrifuged (16,000×*g*, 15 min, 4 °C), and the resulting supernatant was collected for western blot analysis.

Crude synaptosomes were prepared as previously described.^26^ Briefly, brain samples were homogenized in 10% (w/v) ice-cold buffer containing 0.32 M sucrose, 20 mM HEPES pH 7.4, 1 mM EDTA, 1× protease inhibitor cocktail (Roche, Mannheim, Germany), 5 mM NaF, 1 mM Na_3_VO_4_, and 5 mM Na_2_HPO_4_. After centrifugation (800×*g*, 10 min, 4 °C), the supernatant was further centrifuged at 15,300×*g* for 10 min. The supernatant (cytosolic fraction) was removed and the remaining pellets, corresponding to the crude synaptosomal fraction, were resuspended in a lysis buffer (150 mM NaCl, 50 mM Tris–HCl, pH 7.4, 1% Triton X-100, 0.1% SDS, 1× protease inhibitor cocktail, 2 mM EDTA, 5 mM NaF, 1 mM Na_3_VO_4_, and 5 mM Na_2_HPO_4_).

### Western Blot

Sample protein concentrations were measured using the Bio-Rad DC protein assay (Bio-Rad Laboratories, Hercules, CA). Proteins (40 μg) were separated with SDS-PAGE under reducing and denaturing conditions and blotted to a PVDF membrane as previously described.^24^ Membranes were incubated overnight in +4 °C with following primary phosphoprotein antibodies: anti-p-TrkA^Y785^/p-TrkB^Y816^ (#4168; RRID:AB_10620952; 1:000; Cell Signal Technology, Leiden, Netherlands [CST]), anti-p-GSK3β^S9^ (#9336, RRID: AB_331405, 1:1000, CST), anti-p-p70S6K^T421/S424^ (#9204, RRID: AB_2265913, 1:000, CST), anti-p-ERK1/2^T202/Y204^ (p44/42 MAPK; #9106, RRID: AB_331768, 1:1000, CST), and anti-p-Src^Y416^ (#6943, RRID: AB_10013641, 1:1000, CST). For normalization, the following total protein antibodies were used: anti-TrkB (#4603, RRID:AB_2155125, 1:1000, CST), anti-mTrkB (#AF1494, RRID:AB_2155264, 1:1000, R&D Systems), anti-GSK3β (#9315, RRID:AB_490890, CST), anti-p70S6K (#2708, RRID:AB_390722, CST), anti-ERK1/2 (#9102, RRID:AB_330744, 1:1000, CST), anti-β-Actin (#3700, RRID:AB_2242334, 1:5000, CST), and anti-GAPDH (#2118, RRID: AB_561053, 1:1000, CST). The membranes were washed with TBS containing 0.1% Tween (TBST) and incubated with horseradish peroxidase conjugated secondary antibodies (Bio-Rad Laboratories; 1:5000 or 1:10,000 in nonfat dry milk, 1 h, room temperature). Secondary antibodies were visualized using enhanced chemiluminescence (ECL Plus, Thermo Scientific, Vantaa, Finland) for detection using the Biorad ChemiDoc MP camera (Bio-Rad Laboratories).

### Nontargeted Metabolomics

The nontargeted metabolomics method has been described in detail before.^97^ Briefly, weighed tissue samples were extracted using 100 μL of 80% methanol (v/v H_2_O, LC–MS Ultra CHROMASOLV, Fluka) per 10 mg of tissue under sonication. After vortexing and centrifugation (13,000 rpm, 5 min, 4 °C), the supernatant was collected and filtered to HPLC bottles through an Acrodisc CR 4 mm (0.45 μm) filter. Samples were analyzed with a UHPLC-qTOF-MS system (Agilent Technologies) using two different chromatographic techniques, i.e., reversed phase (RP) and hydrophilic interaction (HILIC) chromatography and acquired data in both positive and negative polarity. Welch’s ANOVA was used for statistical analysis. Principal component analysis (PCA) was used for metabolite profiling data to analyze the overall variance between all the samples, and partial least sum of squares discriminant analysis (PLS-DA) was used to calculate VIP values, identifying molecular features explaining the variance between the study groups. Fifteen principal components were needed to explain 95% of the variance in the metabolomics data; therefore, the α level was adjusted to 0.0033 to account for multiple testing. Cohen’s *d* effect sizes were calculated between the treatment and control groups. MassHunter Acquisition (Agilent Technologies, ver.B.04.00) was used for data acquisition, Profinder (Agilent Technologies, ver.B.08.00) was used for feature extraction and peak alignment, the Mass Profiler Professional (MPP, Agilent Technologies, ver.13) for statistics, SIMCA (Umetrics, ver.14.0.0) to perform multivariate analyses, and MS-DIAL (ver.2.90) for metabolite identification.

### Statistical Analysis

The data were primarily analyzed with Student’s unpaired *t*-test and one- or two-way analysis of variance (ANOVA) with Tukey’s post hoc test using Graphpad Prism (v9.51; La Jolla, CA, USA). Comparisons of groups with unequal variances were corrected using Welch’s, Dunnett’s, or Dunnett’s T3 tests, depending on the number of groups and comparisons. When multiple individual pairwise comparisons to their respective control treatments were performed, for example in Figure 1A, no mathematical correction was made for multiple comparisons. The *p* values are described in figure legends, and details of statistical tests and *n* numbers are presented in Table S1.

## Ethics Approval

The animal experiments were carried out in compliance with the European Communities Council Directive of 22 September 2010 (Directive 2010/63/EU), according to the guidelines of the Society for Neuroscience, and approved by the County Administrative Board of Southern Finland (Licenses ESAVI/9793/04.10.07/2016 and ESAVI/5844/2019).

## Supporting Information Available

Pharmacological manipulation of TrkB signaling in the adult brain; despite slowing down cortical activity, atropine does not acutely activate TrkB signaling; medetomidine-induced TrkB signaling occurs ubiquitously in different separated fractions and brain regions; disrupting of hypothermia attenuates the effects of medetomidine and isoflurane on TrkB signaling; changes in blood glucose after administration of amitriptyline; prominently TrkB-activating treatments induce hypothermia; Src family kinase phosphorylation during sedative-state and deep sleep; and noncanonical, sedation-coupled activation of TrkB signaling is associated with reduced ERK1/2 phosphorylation (PDF)
Full statistical tables of main and supplementary figures (XLSX)
