# Supplementary material for: Linking Hypothermia and Altered Metabolism with TrkB Activation
Source: ACS Chem Neurosci. 2023 Aug 8;14(17):3212–25. doi: 10.1021/acschemneuro.3c00350 (PMC10485900; doi:10.1021/acschemneuro.3c00350)
Supplement: Supplementary file 1 — cn3c00350_si_001.pdf [file cn3c00350_si_001.pdf]

Supporting information:

Linking hypothermia and altered metabolism with TrkB activation

Okko Alitalo[1,2], Gemma González-Hernández[1,2#], Marko Rosenholm[1,2,3#], Piia Kohtala[1,2,4], Nobuaki Matsui[5], Heidi Kaastrup Müller[6], Wiebke Theilmann[1], Anders Klein[7,8], Olli Kärkkäinen[9,10], Stanislav Rozov[1,2], Tomi Rantamäki[1,2#] and Samuel Kohtala[1,2,4#*]

[1]Laboratory of Neurotherapeutics, Drug Research Program, Division of Pharmacology and Pharmacotherapy, Faculty of Pharmacy, University of Helsinki, Finland
[2]SleepWell Research Program, Faculty of Medicine, University of Helsinki, Finland
[3]Center for Translational Neuromedicine, Faculty of Health and Medical Sciences, University of Copenhagen, Denmark
[4]Department of Psychiatry, Weill Cornell Medicine, New York, NY, USA
[5]Faculty of Pharmacy, Gifu University of Medical Science, 4-3-3 Nijigaoka, Kani, Gifu, 509-0293, Japan
[6]Translational Neuropsychiatry Unit, Department of Clinical Medicine, Aarhus University, Denmark
[7]Novo Nordisk Foundation Center for Basic Metabolic Research, University of Copenhagen, Denmark
[8]Department of Drug Design & Pharmacology, University of Copenhagen, Denmark
[9]School of Pharmacy, University of Eastern Finland, Kuopio, Finland
[10]Afekta Technologies Ltd., Kuopio, Finland

Short title: Hypothermia and TrkB

[#]equal contribution

*Corresponding authors: Samuel.kohtala@helsinki.fi, tomi.rantamäki@helsinki.fi

Contents

- Figure S1-S8

Supplementary figures

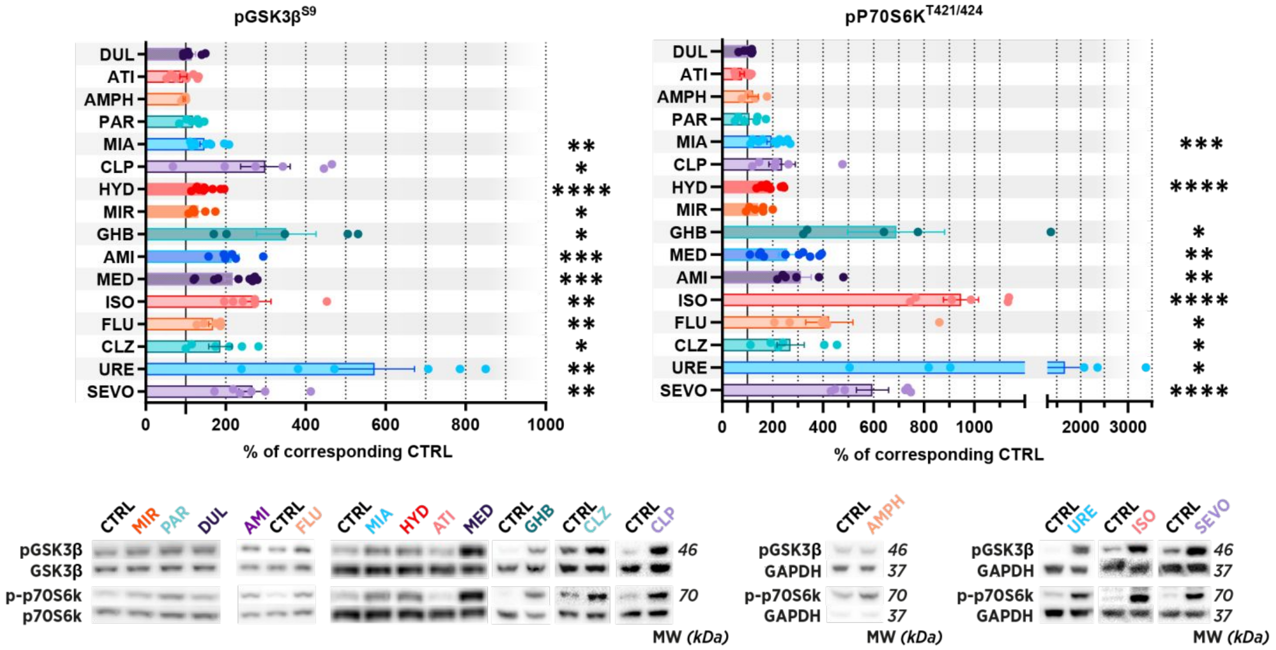

Figure S1. Pharmacological manipulation of TrkB signaling in the adult brain. Diverse sedative-anesthetic agents increased the phosphorylation of GSK3β^{S9} and p70SK6$^{T421/S424}$ in the medial prefrontal cortex of adult mice, whereas non-sedative antidepressants such as paroxetine and stimulant drugs such as amphetamine and atipamezole showed no effects. Phosphoproteins were normalized against corresponding total protein signal and compared to the saline group set to 100%. Control animals for injected pharmacological agents were injected with a vehicle using equal volume and identical route of administration. Control animals for isoflurane and sevoflurane were subjected to pressurized room air. Data are presented as mean ± standard error of mean (S.E.M.). *<0.05, **<0.01, ***<0.001, ****<0.0001 (for statistical analyses and *n* numbers see **Table S1**). Abbreviations: GSK3β, glycogen synthase kinase 3β; p70S6k, ribosomal protein S6 kinase. For the abbreviations and doses of drug treatments, see **Figure 1** or **Table S1**.

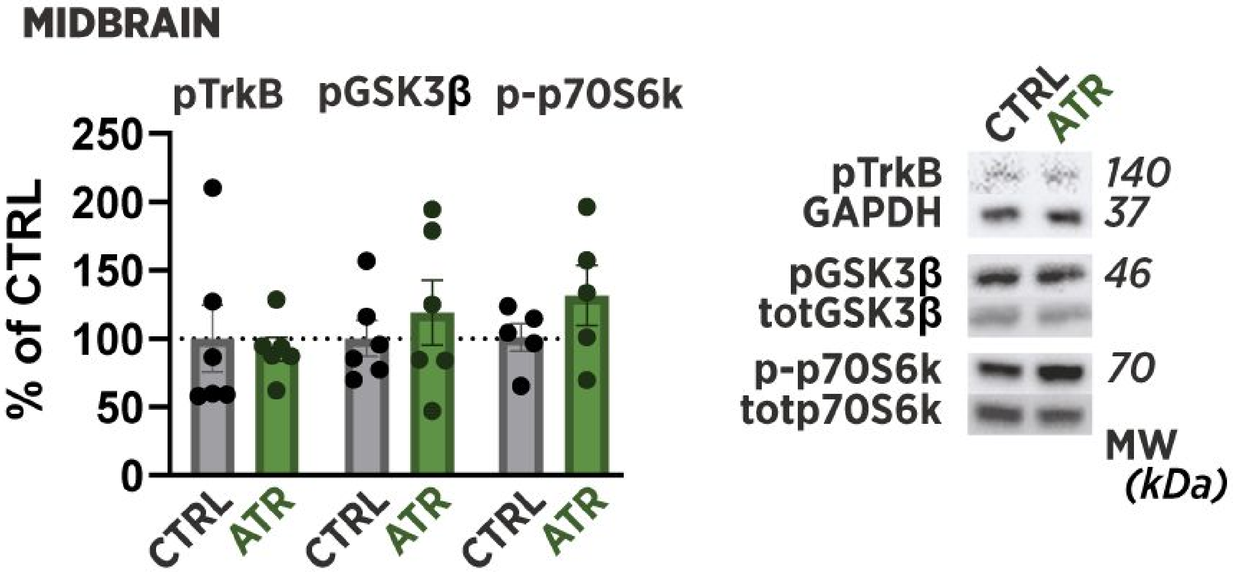

Figure S2. Despite slowing down cortical activity, atropine does not acutely activate TrkB signaling. In comparison to control (CTRL), midbrain samples collected from atropine-treated (ATR; 100 mg/kg, i.p.) animals 30 minutes after injection show negligible activation of $TrkB^{Y816}$, and $GSK3\beta^{S9}$, and $p70SK6^{T421/S424}$. Data are means ± S.E.M (for statistical analyses and *n* numbers see **Table S1**).

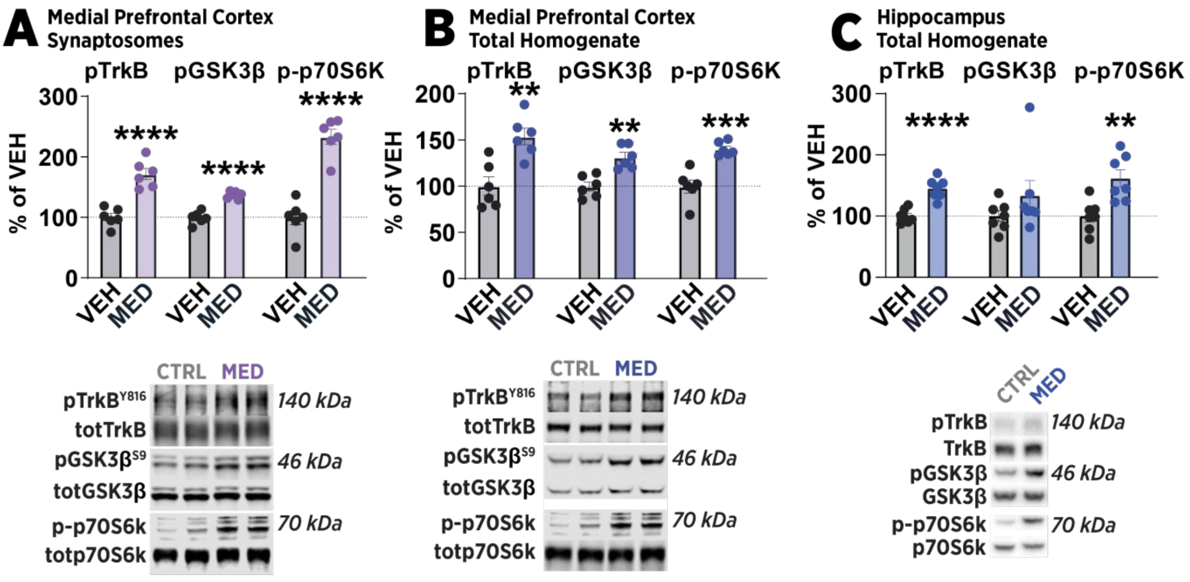

Figure S3. Medetomidine-induced TrkB signaling occurs ubiquitously in different separated fractions and brain regions. Medial prefrontal cortex samples were collected 30 minutes after medetomidine (MED; 0.3 mg/kg) or saline (CTRL) injection (i.p.). Crude isolated synaptosomal fraction (B) and total brain homogenate (B) were prepared as from dissected mouse medial prefrontal cortex as described (Kohtala et al., 2019) for the analysis of TrkB^{Y816}, and GSK3β^{S9} and p70SK6$^{T421/S424}$ phosphorylation. In a similar manner, total homogenates from mouse hippocampus were prepared and analyzed for TrkB^{Y816}, and GSK3 β^{S9} and p70SK6$^{T421/S424}$ phosphorylation (**C**). Phosphoproteins were normalized against corresponding total protein, and the saline group set to 100%. Data are means ± S.E.M. p *<0.05, **<0.01, ***<0.001 (for statistical analyses and *n* numbers see **Table S1**).

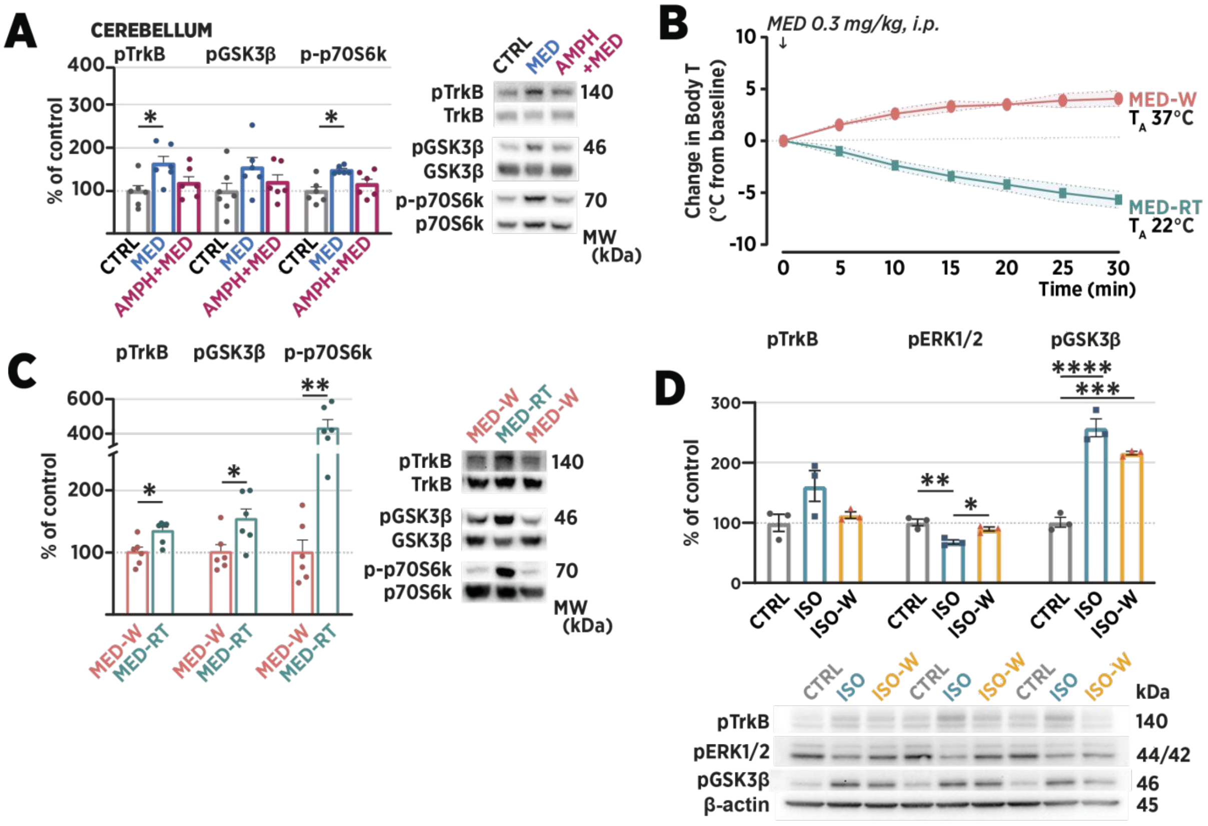

Figure S4. Disrupting of hypothermia attenuates the effects of medetomidine and isoflurane on TrkB signaling. (**A**) The impact of thermogenic amphetamine (AMPH; 10 mg/kg, i.p.) pretreatment on medetomidine (MED; 0.3 mg/kg, i.p.)-induced phosphorylation of TrkB^{Y816}, GSK3β^{S9}, and p70SK6$^{T421/S42}$ in cerebellum. AMPH was administered 15 minutes before MED, and the animals were terminated 30 minutes later. Treatment groups are compared to a sham-treated group (CTRL). (**B**) MED in room temperature (MED-RT) induces prominent hypothermia, but a warm ambient temperature (MED-W; 37 °C) prevents the hypothermic response (**C**) Prevention of the hypothermic response attenuates the MED-induced phosphorylation of TrkB^{Y816}, GSK3β^{S9}, and p70SK6$^{T421/S424}$ in the medial prefrontal cortex samples collected at 30 minutes after injection. (**D**) Maintaining the mice in warm ambient temperature attenuates the phosphorylation of TrkB^{Y816} and GSK3β^{S9} induced by isoflurane (ISO) anesthesia. Maintaining the temperature (ISO-W) also prevents the isoflurane-induced decrease in ERK1/2$^{T202/Y204}$ phosphorylation. Phosphoproteins were normalized against corresponding total protein, and the control group set to 100%. Data are means + S.E.M. *<0.05, **<0.01, ***<0.001, ****<0.0001 (for statistical analyses and *n* numbers see **Table S1**).

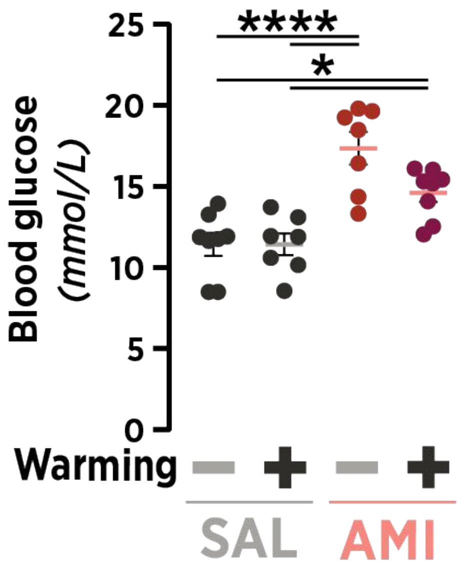

Figure S5. Changes in blood glucose after administration of Amitriptyline. Amitriptyline (AMI; 20 mg/kg, i.p.) increases terminal blood glucose measured at 30 minutes after injection. The effect is attenuated, but not completely abolished, by holding the animals in an incubator (T_A 35°C) after injection until termination, instead of common laboratory environment (T_A 22°C). Control animals were treated with equal volume of saline. Data are means + S.E.M. *<0.05, ****<0.0001 (for statistical analyses and *n* numbers see **Table S1**).

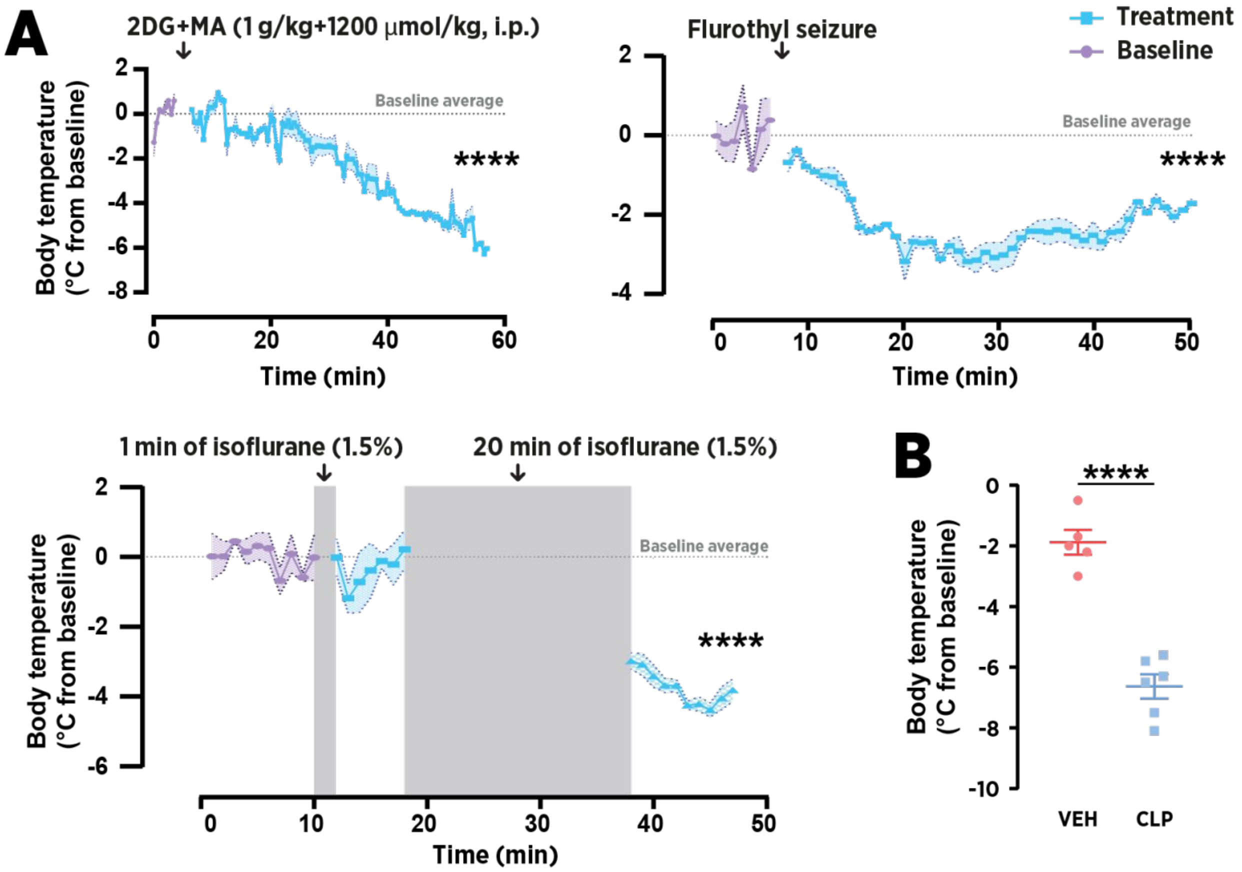

Figure S6. Prominently TrkB-activating treatments induce hypothermia. (**A**) FLIR camera screening of effects of different drugs on body temperature in unrestrained animals. Temperature data was recorded after administration of treatments on small cohort of animals (n=2–3). Isoflurane (1.5%) was administered in a separate chamber and the animals were moved to the imaging chamber separately, resulting in the apparent gradual increase of the temperature during the recording periods. The average baseline temperature was statistically different with all post-treatment recordings, except for the one-minute isoflurane anesthesia. (**B**) Rectal temperature measurement at 30 minutes after injection demonstrates prominent hypothermic effect of chlorpromazine (CLP; 10 mg/kg, i.p.). Data are means + S.E.M. p = ****<0.0001 (for statistical analyses and *n* numbers see **Table S1**).

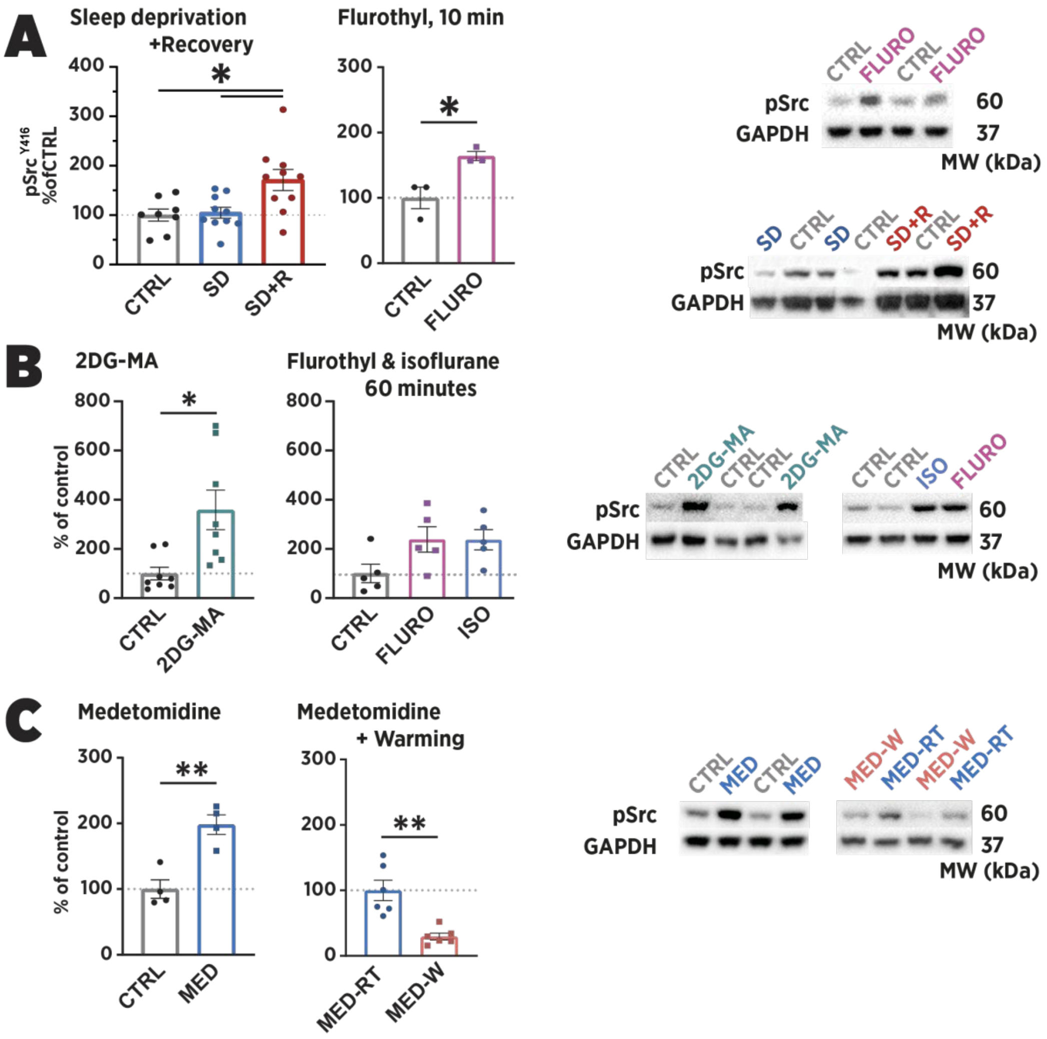

Figure S7. Src family kinase phosphorylation during sedative-state and deep sleep. (**A**) Src family kinases are phosphorylated at Y416 residue in the medial prefrontal cortex samples collected in the early phase of the homeostatic recovery. Phosphorylation at the end of sleep deprivation (SD) is unaffected, while 15 minutes of recovery sleep (SD+R) shows increase. Similarly, Src is activated 10 after the cessation of flurothyl (FLURO) seizure. (**B**) Sustained metabolic inhibition using 2-deoxyglucose and mercaptoacetate (2DG-MA; 1 g/kg and 1200 µmol/kg, respectively, i.p.) shows marked increase in the phosphorylation of Src^{Y416} in samples collected at 45 minutes after injection. 1 hour after FLURO seizure or continuous isoflurane (ISO; 1.5 %) anesthesia, more variability in pSrc ^{Y416} is observed. In full blots incubated with the same pSrc ^{Y416} antibody, clear regulation associated with the treatment is observed in an uncharacterized band of approximately 90 kDa molecular weight. (**C**) The activation of Src kinase, along with TrkB signaling, is temperature-dependent. At 30 minutes, medetomidine (MED; 0.3 mg/kg, i.p.) induces prominent phosphorylation of pSrc ^{Y416}, which is blunted by keeping the animals in an incubator (37 ± 1°C). The control animals for injected treatments received physiological saline (CTRL), while inhaled treatments were controlled using pressurized room air (SHAM). Phosphoproteins were normalized against corresponding total protein, and the control group set to 100%. Data are means + S.E.M. *<0.05, **<0.01 (for statistical analyses and *n* numbers see **Table S1**).

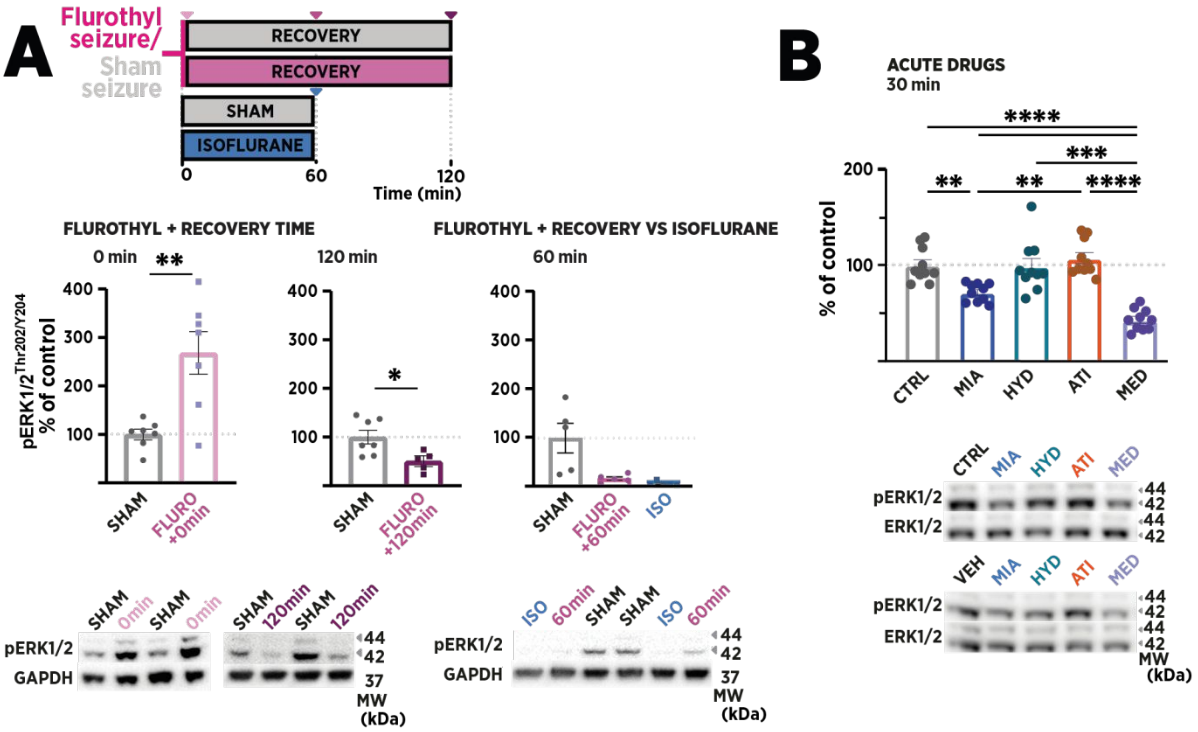

Figure S8. Non-canonical, sedation-coupled activation of TrkB signaling is associated with reduced ERK1/2 phosphorylation. (**A**) Extracellular signal-regulated kinase (ERK1/2; p44/42 MAPK) is phosphorylated in T202/Y204 residues during acute excitatory stimuli such as flurothyl seizure, during which phosphorylation of $TrkB^{Y816}$, $GSK3\beta^{S9}$, and $p70SK6^{T421/S424}$ is unaffected. However, as animals become sedated when the excitatory stimulus subsides, the phosphorylation of $ERK1/2^{T202/Y204}$ is rapidly downregulated to a fraction of the baseline level. Analogous to the postictal sedative state, direct sedation with isoflurane (ISO) or other diverse sedative-anesthetic drugs (**B**) reduces $ERK1/2^{T202/Y204}$ phosphorylation while increasing the phosphorylation of $TrkB^{Y816}$, $GSK3\beta^{S9}$, and $p70SK6^{T421/S424}$. Drugs, doses, and routes administered in **B** were: mianserin (MIA; 20 mg/kg, s.c.), hydroxyzine (HYD; 50 mg/kg, s.c.), atipamezole (ATI; 1 mg/kg, s.c.), and medetomidine (MED; 0.05 mg/kg, i.p.). Control animals for injected pharmacological agents were administered vehicle (VEH; physiological saline) in equal volume. Control animals for flurothyl and isoflurane were subjected to sham treatments (SHAM) consisting of brief holding in induction chamber and pressurized room air, respectively. Data are means ± S.E.M. p * < 0.05, ** < 0.01, *** < 0.001, **** < 0.0001 (for statistical analyses and *n* numbers see **Table S1**).
